# Slosh Simulation in a Computer Model of Canine Syringomyelia

**DOI:** 10.3390/life11101083

**Published:** 2021-10-14

**Authors:** Srdjan Cirovic, Clare Rusbridge

**Affiliations:** 1 Department of Mechanical Engineering Sciences, University of Surrey, Guildford GU2 7XH, UK; 2 The School of Veterinary Medicine, University of Surrey, Guildford GU2 7AL, UK; c.rusbridge@surrey.ac.uk; 3 Fitzpatrick Referrals Orthopedics and Neurology, Halfway Lane, Eashing, Godalming GU7 2QQ, UK

**Keywords:** Valsalva maneuver, Chiari malformation, pathophysiology syringomyelia, biomechanics, Bernard Williams hypothesis, cavalier King Charles spaniel, cerebrospinal fluid

## Abstract

The exact pathogenesis of syringomyelia is unknown. Epidural venous distention during raised intrathoracic pressure (Valsalva) may cause impulsive movement of fluid (“slosh”) within the syrinx. Such a slosh mechanism is a proposed cause of syrinx dissection into spinal cord parenchyma resulting in craniocaudal propagation of the cavity. We sought to test the “slosh” hypothesis by epidural excitation of CSF pulse in a computer model of canine syringomyelia. Our previously developed canine syringomyelia computer model was modified to include an epidural pressure pulse. Simulations were run for: cord free of cavities; cord with small syringes at different locations; and cord with a syrinx that was progressively expanding caudally. If small syringes are present, there are peaks of stress at those locations. This effect is most pronounced at the locations at which syringes initially form. When a syrinx is expanding caudally, the peak stress is typically at the caudal end of the syrinx. However, when the syrinx reaches the lumbar region; the stress becomes moderate. The findings support the “slosh” hypothesis, suggesting that small cervical syringes may propagate caudally. However, when the syrinx is large, there is less focal stress, which may explain why a syrinx can rapidly expand but then remain unchanged in shape over years.

## 1. Introduction

Syringomyelia was first described by Stephanus in 1545; however, over 400 years later, the mechanism by which the spinal cord cavities (syrinx; syringes) form and fill with fluid is still debated. Syringomyelia occurs secondary to cerebrospinal fluid (CSF) channel obstruction with clinical signs of chronic pain, sensory and motor deficits with disability [1,2]. In humans, the compromise in patient quality of life is comparable with that of patients with heart failure [3]. Disruption of CSF flow at the craniocervical junction increases risk of syringomyelia. Morphological changes to the skull, cervical vertebrae, and brain-to-cranial cavity ratio commonly referred to as Chiari malformation can alter dynamics of CSF flow at the craniocervical junction and is the most common cause of syringomyelia. Chiari malformation and syringomyelia affect both humans and animals. Certain brachycephalic toy breed dogs have exceptionally high prevalence of the disorder with one breed, the cavalier King Charles spaniel (CKCS), having a syringomyelia lifetime risk of 70% [4]. Purebred dogs are bred to conform to a certain shape, size and color, and such selections ensure that the conformational traits predisposing this complex trait are similar within that population in comparison to humans where the phenotype is variable. This spontaneously occurring canine model of human Chiari type 1 and 0 malformation provides a unique opportunity for understanding the pathogenesis of syringomyelia, which is believed to be the same in humans and in animals. Animal (rat, sheep) models of induced syringomyelia have been used extensively to gain valuable information on various aspects of syringomyelia, e.g., the nature and the origin of the fluid in syringes [5,6,7]. As the medical interventions used to manage Chiari malformation and syringomyelia in humans and animals are similar, the insights gained from animal studies have direct relevance for the clinical (surgical) treatment of human patients. However, failure of resolution of syringomyelia post-operatively is reported in 10–40% of humans and 100% of dogs [8,9]. The lack of in-depth understanding of the origins of the condition is one of the reasons for the limited success of currently available treatments [10,11].

Currently, the development of theories on syrinx formation strongly relies on in silico (computer) models of the human spinal CSF space. These models can capture spatial and temporal variation of parameters such as CSF pressure, CSF velocity, and mechanical stress in the cord tissue [12,13,14,15,16,17,18,19,20,21,22,23,24,25,26,27,28,29,30,31]. From the results of such studies, it has been proposed that abnormal CSF flow causes syrinx formation via an arterial pulse-driven “perivascular pump” [12,13,14,15,25,26,27], and via excessive repetitive stressing of the spinal cord tissue [16,29,31]. When a syrinx is already present, it is possible that it enlarges through a “slosh” mechanism [32]. First proposed by Bernard Williams in 1980, this hypothesis of syrinx propagation suggests that epidural venous distention during Valsalva maneuvers causes impulsive movement of fluid (“slosh”) within the syrinx [32]. Valsalva maneuvers describe raised intrathoracic pressure when expiring against a closed glottis, for example, coughing sneezing or straining. During Valsalva maneuvers, blood is shifted from thorax to the epidural veins, which compress the dura and generates CSF motion [33,34]. When the CSF in the subarachnoid space (SAS) flows past a syrinx it causes sloshing of the fluid in the cavity and straining of the spinal cord tissue at the syrinx ends. Williams defines slosh as a situation where the CSF in the SAS is set in motion by a Valsalva maneuver and compresses the syrinx at one end. This forces the fluid to the other end of the cavity, which then becomes extended [35]. Bertram et al. [30] examined the slosh mechanism using an idealized model of the human spine and found that CSF flow generated by the compression of the dura may cause elevated stress at the caudal end of an existing syrinx. Other analytical models suggest that the syrinx expansion may be governed by an inherent “homeostatic” mechanism that works to alleviate abnormally high stress in the spinal cord [16]; an initial syrinx expands until stress in the cord returns to some normal “baseline” values.

Animal computer models of syringomyelia are much less frequent than human models. Yet they can be useful, especially when representing species with a high incidence of the condition, and a highly specific pattern of cavity formation. We previously developed a computer (finite element) model of canine spinal cavity to investigate possible causes of syrinx formation [36]. The model geometry was extracted from magnetic resonance imaging (MRI) scans of a CKCS with a Chiari-like malformation and a fully developed syrinx. Cardiac pulse excitation of CSF motion was simulated for a fully patent and a fully occluded foramen magnum, with the latter representing the extreme case of CSF pathway obstruction due to Chiari-like malformation. When the foramen magnum was fully patent, the model predicted mechanical stress in the spinal cord to be of the same magnitude as the CSF pressure (order of magnitude of 10^2^ Pa). When the foramen magnum was obstructed, the stress values in the cranial cervical region and the cervico-thoracic junction increased by nearly 100%; these are also the regions where initial syringes form in dogs [37,38]. While the absolute values of mechanical stress predicted by the model were probably too low to cause any immediate damage to the spinal cord tissue, abnormally high stress was clearly correlated with typical anatomical locations at which syringes initially form. Without trying to explain the exact mechanism(s) for syrinx formation and growth, we hypothesize that they can be associated with the stress in the spinal cord being significantly elevated from the values that would normally be generated by common excitations such as the cardiac pulse and filling of the epidural veins. In this study, we build on the above hypothesis to examine the situation where small initial syringes have already formed in the spinal cord tissue. The focus is on the excitation of CSF movement caused by the filling of epidural veins, which can potentially generate the “slosh” effect leading to syrinx expansion.

## 2. Materials and Methods

The CSF dynamics in a canine spine was investigated using a finite element model. The finite element method is an approximate method for solving complex engineering problems. In this method, the domain is “discretized” by breaking it into a large number of sub-components (e.g., hexagons or tetrahedrons) called “elements”. For each element in the model, simplifying approximations are applied to the equations governing the behavior of the system examined. The resulting set of equations involving all the elements is solved using computers to yield quantities such as displacement, speed, strain, and stress in each element.

### 2.1. The Finite Element Model

**Figure 1:**
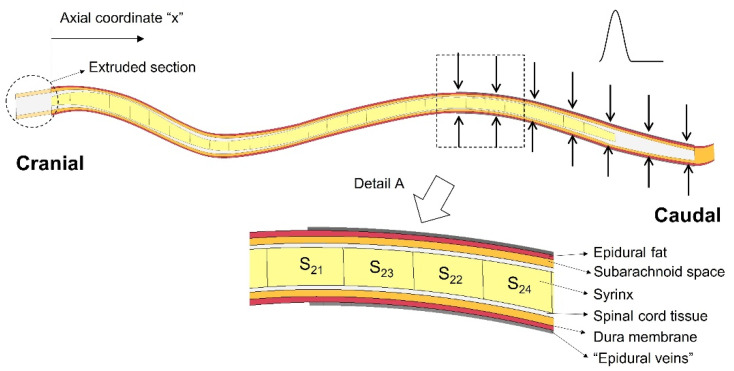
The finite element model of the canine spinal cavity (mid-sagittal view). The arrows denote the area at which the epidural pressure was applied. Detail A shows the anatomical layers included in the model, as well as 1 cm long sections (21–24) of the syrinx.

The finite element model of the spinal cavity used in this study is based on the model developed by Cirovic et al. [36], with modifications introduced in order to simulate expansion of the epidural venous plexus and to examine various scenarios of syrinx placement and syrinx expansion. The model geometry is semi-idealized, in the sense that it is derived from an actual anatomically accurate geometry, which was then simplified. The detailed description of the geometry generation is given in Cirovic et al. [36], and here it will be presented only briefly. The main focus will be on the modifications introduced for the purpose of the current study. The geometry was extracted from magnetic resonance imaging (MRI) scans of a CKCS dog with a Chiari-like malformation and a fully developed syrinx extending between second cervical (C_2_) and third lumbar (L_3_) vertebrae. The distance between the cranial and caudal end of the syrinx was 285 mm. The following anatomical layers were included in the model: the epidural space, SAS, the spinal cord, and the syrinx cavity (see detail A in Figure 1). The dura was represented as a 1 mm thick shell between the SAS and the epidural space. To reduce the complexity of the model and create a fully hexagonal finite element mesh, all the components of the spinal cavity were simplified to have a circular section in the transverse plane. The “radii” of all anatomical layers were determined to match their actual cross-sectional areas from the MRI scans. Their values are tabulated in Cirovic et al. [36]. They can also be found in the Supplementary Materials (“Geometric parameters of the model.xlsx”). Furthermore, the spine was assumed to be symmetric about the mid-sagittal plane, such that only one half of the spinal cavity is represented in the model. The cranial end of the model (spinal cord and SAS) was extended by 15 mm (“extruded section” in Figure 1). This was performed to remove spurious stress concentrations, which would form in the cranial end of the spinal cord if it was constrained directly at C_2_.

For this study, we also created a version of a model geometry in which the syrinx radius is 70% of the cord radius throughout its length. This was for the purpose of simulating the effect of small, isolated syringes at different locations in the spine (as discussed in the next paragraph). In addition, the actual syrinx was segmented into 28 segments (twenty-seven 10 mm segments and one 15 mm segment at the caudal end). Each segment can be assigned properties of either fluid or spinal cord tissue material. This allows different patterns of syrinx size and location to be explored using essentially the same model geometry. The outer surface of the model (epidural space and SAS in the extruded section) was constrained from moving in any direction, except in the region where the epidural pressure was applied. The cranial end of the spinal cord (extruded) was constrained from moving in any direction, and the caudal end of the cord was constrained from moving in the cranial-to-caudal direction. Symmetry was imposed in the sagittal plane. The epidural veins were represented in the following way: A 0.5 mm thick control volume was created around the epidural layer between x = 205 mm and x = 325 mm, where x is a cranial-to-caudal axis starting at the cranial end of the syrinx. This region is indicated with arrows in Figure 1, and the control volume is shown as “epidural veins” in the detail A. The pressure in the control volume is related to the inflow and outflow of fluid (venous blood) and to the stiffness of the structures defining the volume. All the surfaces defining the control volume were rigid (vertebrae), except for the outer surface of the epidural fat on which the epidural veins act. The segment of the spine where the epidural excitation was exerted was chosen following the approach used for the human computer model by Bertram et al. [30]. It was also consistent with the human experimental data reported by Williams et al. [33], which indicate that the epidural pulse is generated at the caudal end of the spine. Cerebrospinal fluid in the SAS and the fluid in the cavities were modeled as nearly incompressible viscous fluids and the remaining components were modeled as linear elastic solids. The material properties of the tissues and fluids were as in Cirovic et al. [36] and they are summarized in Table 1. A Lagrangian formulation was used for the solid parts in the model and an Arbitrary Lagrangian Eulerian (ALE) formulation was used for the fluid parts. All simulations were performed using the LS-DYNA finite element package for dynamic simulations (LS-DYNA release R11, Livermore Software Technology Corp., Livermore, CA). The solution is obtained by solving the 3D momentum equation:

(1) $$\sigma_{ij,j}+\rho f_{i}=\rho {\ddot{x}}_{i}$$

For the specified boundary and initial conditions, here, *σ_ij_* is the Cauchy stress, *ρ* is the current density, *f* is the body force density, $\ddot{x}$ is acceleration, and the comma denotes covariant differentiation. The domain is discretized using linear hexagonal elements with reduced integration. An explicit second-order central scheme is used for time integration. For the fluid parts the material is first deformed in a Lagrangian step, and then state variables are advected onto the (moving) ALE mesh [39].

### 2.2. Simulations

The epidural excitation was simulated by prescribing a hypothetical pressure input in the control volume (epidural veins). The LS-DYNA built-in function “fluid-filled (air)bag” implements the assigned pressure and calculates the appropriate influx of the nearly incompressible fluid (venous blood) into the control volume. The input pressure waveform was set to loosely follow human experimental data from Williams at al. [33,34] in terms of amplitude and duration of the pulse. The pulse duration was set at 0.2 s with the amplitude of 6000 Pa (approximately 45 mmHg). A hypothetical analytical function p(t)=3000[1−cos(10πt)] was used to define epidural pressure (*p*) for time *t* < 0.2 s. Otherwise, the epidural pressure was set to zero.

**Figure 2:**
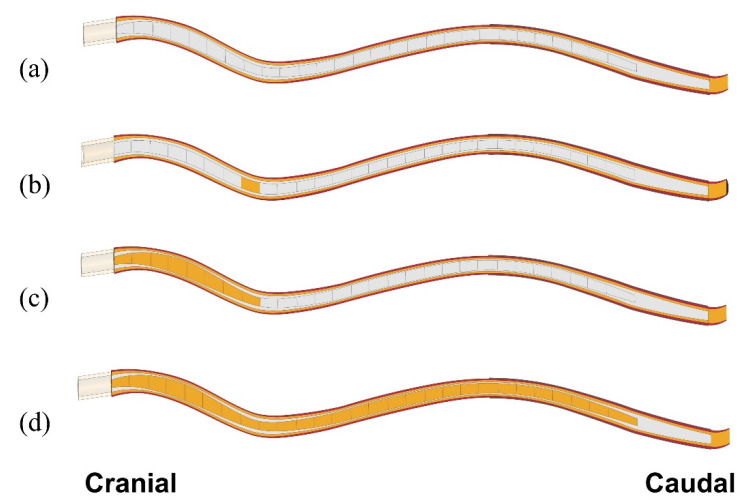
Examples of model configurations used in the study. (**a**) Spinal cord free of syringes. (**b**) One small (10 mm long) syrinx starting at 70 mm from the cranial end of the cord; syrinx radius is 70% of the cord radius (S_8_). (**c**) An 80 mm long syrinx stretching from the cranial end (S_1–8_); syrinx radius is determined from MRI data. (**d**) Full length of the syrinx (S_1–28_).; radii determined from MRI data.

Simulations were performed to examine how the presence of syringes affects critical parameters of CSF/dynamics, and how this may be related to the expansion of cavities. The focus was primarily on the mechanical stress in the spinal cord tissue. Two scenarios were considered: In the first scenario, we examined the effect of small (10 mm long) initial syringes being present at different locations in the spinal cord with the rest of the cord remaining intact. This was to examine whether “slosh” could be a mechanism causing expansion of small initial isolated syringes. The 280 mm long stretch of the cord affected by the actual syrinx was considered as the region in which initial small syringes may form. Twenty-eight separate model configurations were constructed to cover the region of interest in 10 mm increments. We refer to these small syringes, and the corresponding model configurations as “S_i_“, where “S” stands for syrinx and “i” is the distance (in cm) between the caudal end of the syrinx and the cranial end of the spinal cord. Thus, a small syrinx stretching between 70 and 80 mm from the cranial end is referred to as ”S_8_” (Figure 2b). For this set of simulations, the syrinx radius was always kept at 70% of the spinal cord radius (the average ratio for the actual syrinx and spinal cord). In the second scenario, we examined the effect of syrinx expansion on the stress in the spinal cord. Only one possibility for syrinx expansion was considered, in which the syrinx initially formed at the cranial end of the spinal cord and then grew steadily toward the caudal end until it reached its full length. Syrinx growth was considered in 10 mm increments; thus, 28 separate model configurations were constructed. Here, the syrinx radius varied according to actual data obtained from the MRI scans. Since different configurations were effectively obtained by stacking 10 mm syrinx segments together, they are referred to as S_1_–j, where j is the number of the caudal-most syrinx increment. Thus, a syrinx stretching 80 mm caudally is referred to as S_1–8_ (Figure 2c), and the fully developed syrinx is referred to as S_1-28_. Simulations were also run for a model with cord free of any syringes to serve as a benchmark for “nominal” parameter values (Figure 2a). In total, 57 model configurations were considered in the study.

### 2.3. Data Processing

**Figure 3:**
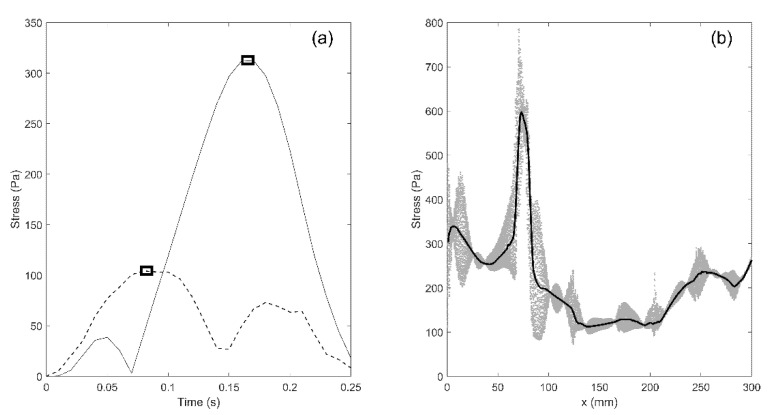
Processing of the data for mechanical stress in the spinal cord. (**a**) traces of the von Mises stress in two elements in the spinal cords. The symbols indicate the peak values recorded over the duration of the simulated event. (**b**) The scatter gives the peak values of stress for all the elements of the spinal cord. The thick black line gives the median values of peak stress for 1 mm thick slices of the spinal cord.

The simulations yield results in terms of movement/deformation for all components of the model, pressure in the fluid compartments (SAS, syringes), and mechanical stress. All these parameters can be traced for any part of the model and at any moment in time. For example, the distribution of von Mises (equivalent) stress in the spinal cord can be displayed as a color map which changes over time. In addition, traces of stress versus time can be plotted for each element in the model. However, we are mainly interested in mapping the distribution of peak stress in the spinal cord at different locations, regardless of the time at which the peak values occurred. To achieve that, the peak value of von Mises stress was determined for each element in the cord and that value was assigned to the location occupied by the element. The procedure is illustrated in Figure 3a. The solid and broken lines display traces of von Mises stress in two elements of the spinal cord. The square symbols are the peak values of stress for the two elements. They do not occur at the same moment of time, but regardless, they were assigned to the points in the cord where the elements were located to produce the map of maximal stress distribution.

It is also useful to define a one-dimensional parameter that can be used to quantify stress magnitude distribution along the length of the spine. In order to achieve that, the results were processed in the following way: the spinal cord was divided in 1 mm transverse slices; next, the median value of peak von Mises stress for all the elements within a slice was calculated and that value was used as a measure of stress in the slice. The procedure is illustrated in Figure 3b. The *x*-axis is the cranial-to-caudal axis shown in Figure 1. The scatter gives peak values of von Mises stress for all the elements comprising the spinal cord. The thick black line gives median values of peak von Mises stresses for all 1 mm thick axial slices between the cranial and caudal end of the spinal cord.

## 3. Results

### 3.1. General Observations

#### 3.1.1. Pressure in the SAS and Syringes

**Figure 4:**
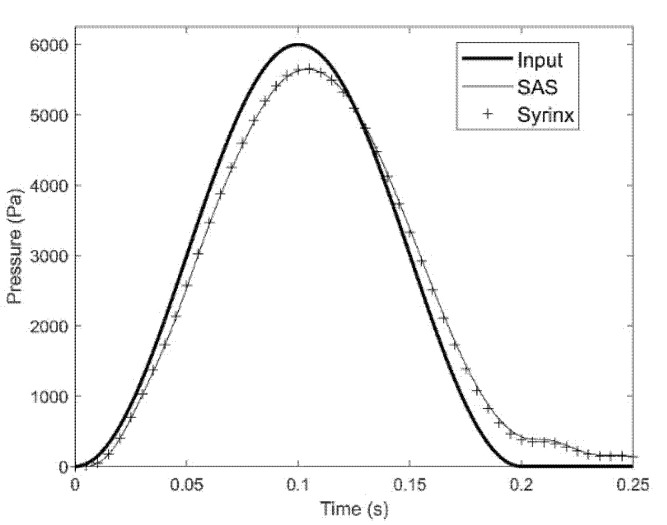
Input pressure waveform (thick line), pressure trace in the SAS near a small syrinx (thin line), and pressure trace in the syrinx (symbol). The results were obtained for model configuration with a single small syrinx (S_8_). Time is measured from the onset of the pressure excitation in the epidural space.

Pressure exerted on the epidural space propagates through the spinal cavity with the shape and magnitude of the waveform remaining largely undisturbed. Figure 4 shows the trace of the input pressure in the epidural veins and the traces of the pressure recorded in the SAS and the syrinx of the S_8_ model configuration at x = 75 mm. It can be seen from the figure that the pressure reaches the cervical part of the spine with the waveform remaining almost undisturbed. There is only a small drop in the amplitude as the pressure is transmitted from the epidural space to the SAS across the dura membrane. Pressures in the syrinx and the surrounding SAS are almost identical, meaning that pressure gradient across the spinal cord is low.

#### 3.1.2. Movement in the Anatomical Layers

**Figure 5:**
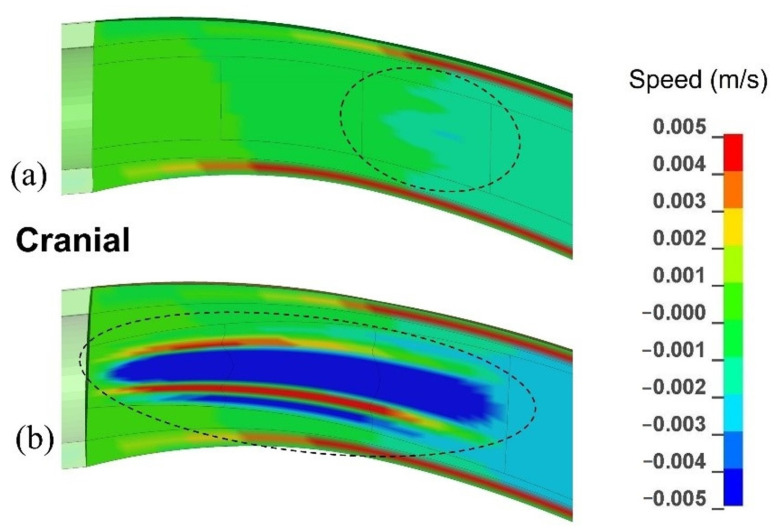
The distribution of axial (caudal-to-cranial) velocity in the SAS, spinal cord, syrinx for the cranial portion of the model. The displayed results are for 0.08 s after the onset of the pressure pulse in the epidural space. (**a**) The model configuration with one small syrinx stretching from x = 20 mm to x = 30 mm (S_3_). (**b**) The model configuration with an enlarging syrinx stretching from x = 0 to x = 30 mm (S_1–3_).

The pressure wave generated movements of all the layers in the spinal cavity. The order of magnitude of velocity in all anatomical layers was 10^−3^ m/s. Figure 5 gives the distribution of velocity in the caudal-to-cranial direction in the cervical region of the SAS, spinal cord, and the syrinx. Figure 5a corresponds to the model configuration with a single small syrinx between x = 20 mm and x = 30 mm (S_3_), whereas Figure 5b corresponds to an enlarging syrinx stretching between x = 0 and x = 30 mm (S_1–3_). For the S_3_ configuration, the fluid in the syrinx and the surrounding spinal cord tissue move together. However, different regions of the cord and syrinx move at different speeds which causes stretching and compression of the syrinx. For the S_1-3_ configuration, the movement of the fluid in the 30 mm long syrinx is more complex and more pronounced. The fluid no longer moves together with the cord. Faster caudal movement in the central part of the syrinx dominates, which indicates that the bulk of the fluid is shifting to the caudal end of the syrinx. However, the movement is not unidirectional, and there is a small region of syrinx fluid around the central core that is moving cranially (red zones in Figure 5b. In both cases, CSF in the SAS moves cranially and considerably faster than the cord.

#### 3.1.3. Patterns of the Mechanical Stress in the Spinal Cord

**Figure 6:**
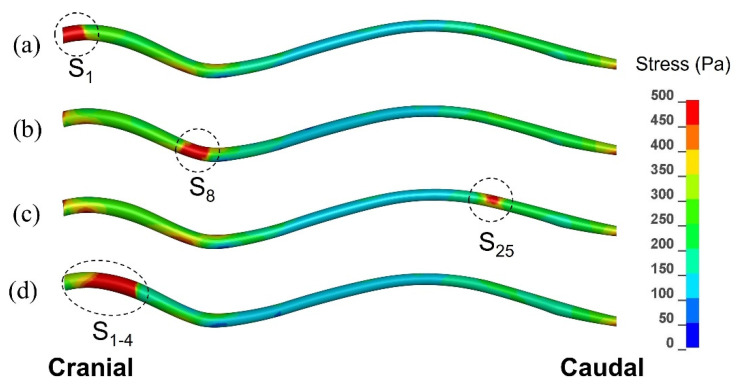
Peak von Mises stress in the spinal cord recorded in each element over the duration of the simulated event, for three configurations with a small syrinx, and one with an expanding syrinx (lateral view). (**a**) Small syrinx stretching from x = 0 to x = 10 mm (S_1_). (**b**) Small syrinx stretching from x = 70 mm to x = 80 mm (S_8_). (**c**) Small syrinx stretching from x = 240 mm to x = 250 mm (S_25_). (**d**) An expanding syrinx stretching from x = 0 to x = 40 mm (S_1–4_).

Figure 6 displays the distribution of peak von Mises stress recorded in each element of the spinal cord over the whole duration of the simulated event. As explained in the previous section, this is not stress distribution at any specific moment of time, but rather a map of the maximal stress experienced at each point in the spinal cord. The results are shown for three scenarios involving a single small syrinx (S_1_, S_8_, and S_25_), and for one scenario involving an enlarging syrinx (S_1–4_). The stress values were of the order of 10^2^ Pa, i.e., one order of magnitude lower than the pressure in the epidural space, SAS, and the syringes. From the figure it can be clearly seen that there were highly localized regions of stress which closely corresponded to the locations of syringes. The maximum stress values were 780 Pa for S_1_, 850 Pa for S_8_, 535 Pa for S_25_ and 920 Pa for S_1–4_. Elsewhere, the stress distribution was similar for all scenarios shown in Figure 2; higher stress was recorded in the cervical, followed by the lumbar region, whereas the thoracic region experienced the lowest stress. It can be also seen from the figure that the extent to which the stress was influenced by a small syrinx depended on the location of the syrinx; S_1_ and S_8_ have stronger effect on the stress distribution than S_25_.

### 3.2. Simulation Results for Isolated Syringes and for the Expanding Syrinx

#### 3.2.1. Small, Isolated Syringes

**Figure 7:**
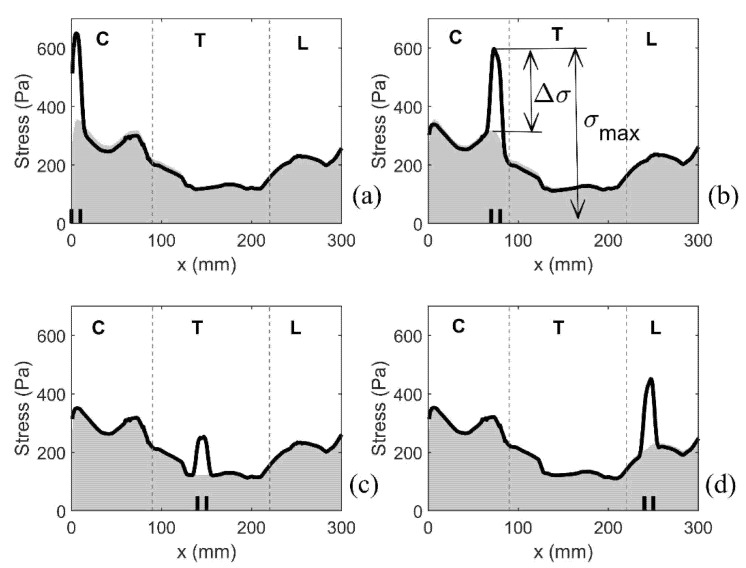
Distribution of the stress along the length of the spinal cord. Coordinate x corresponds to the axial position measured in the cranio-caudal direction (see Figure 1). The stress is quantified via the median values of the peak Von Mises stress recorded in 1 mm-thick slices over the duration of the simulated event. Shading gives the stress distribution for the spinal cord free of syringes. Thick black lines display results for the following four model configurations: (**a**) S_1_, (**b**) S_8_, (**c**) S_15_, and (**d**) S_25_. Short vertical lines on the horizontal axis denote cranial and caudal ends of the syrinx. Vertical broken lines divide the cord into cervical (C), thoracic (T), and lumbar (L) segment.

As previously discussed, the median value of the peak von Mises stress in 1 mm thick slices of the spinal cord was used as a measure of stress magnitude along the length of the spinal cord. Figure 7 shows the typical results for four different configurations with a single small syrinx. The (top edge of the) shaded area gives the results for the cord free of syringes, whereas the thick black lines refer to model configurations S_1_, S_8_, S_15_ and S_25_. For the cord free of syringes, the stress remains below 360 Pa. The cervical region experiences the highest stress, followed by the lumbar region. The lowest stress is in the thoracic region. The presence of syringes results in a sharp increase of stress values. That increase is highly localized, affecting only the immediate vicinity of the syrinx. For the remainder of the cord, the stress is virtually unaffected. The increase in stress from the referent values are up to 100% and they strongly depend on the syrinx location. Thus, both the increase in stress and the absolute stress values are the most pronounced in the cervical region and they are more modest in the thoracic region.

**Figure 8:**
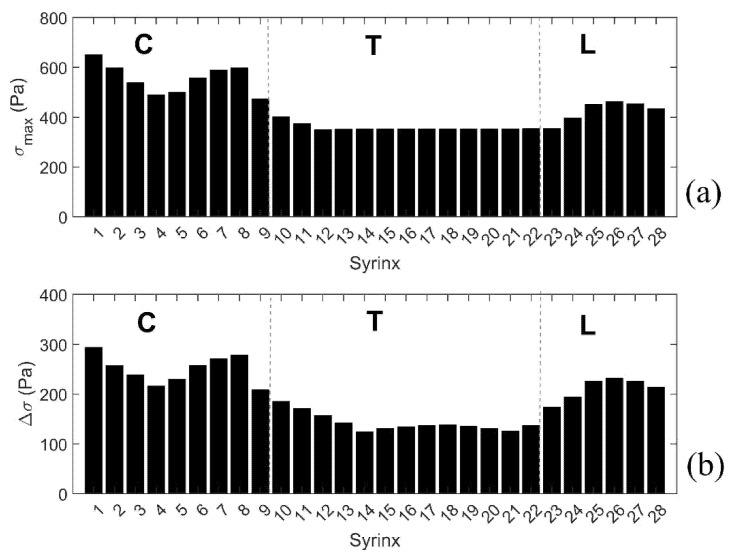
Peak stresses, and stress increases from the baseline values for the 28 positions of the small initial syringes. (**a**) Peak value of the median Von Mises stress (σ_max_). (**b**) Increase of the median Von Mises stress from the baseline values (Δσ). Vertical broken lines divide the cord into cervical (C), thoracic (T), and lumbar (L) segment.

To further summarize the results for all models with a single small syrinx, we focus on two parameters shown graphically in Figure 7b. The first parameter σ_max_ gives the peak value of the (median von Mises) stress in the cord at the location of a syrinx. The second parameter Δσ gives the increase of the (median von Mises) stress from the baseline value (spinal cord without syringes). These parameters can be loosely interpreted as measures of the sensitivity of the spinal cord to the presence of small syringes at different locations along its length (in terms of elevated stress). The results for all 28 syrinx positions are given as bar graphs in Figure 8. From Figure 8a, it can be seen that the highest values of σ_max_ are in the cervical region, and especially for S_1_ (650 Pa), S_7_ (590 Pa) and S_8_ (600 Pa). The values of Δσ are much lower (below 400 Pa) in the thoracic region and become slightly higher in the lumbar region. The values of Δσ for the 28 syrinx locations are shown in Figure 8b. It can be seen from the figure that the pattern is similar to that of σ_max_, with highest values of just below 300 Pa occurring at S_1_ and S_8_, the lowest values (under 200 Pa) occurring in the thoracic region, and with a rebound of Δσ occurring in the lumbar region. In terms of percentage or stress increase from the baseline values, this ranged between 54% and 100%, with a median value of 80%.

#### 3.2.2. Expanding Syrinx

**Figure 9:**
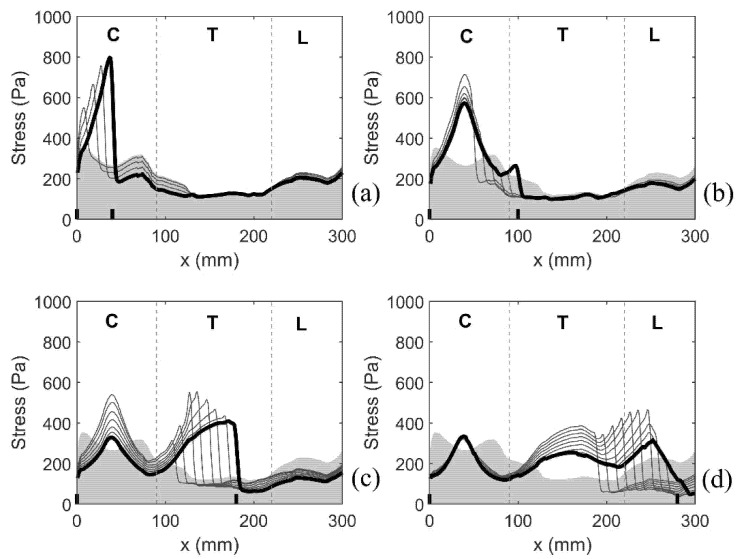
Distribution of von Mises stress along the length of the spinal cord as the syrinx is expanding from the cranial toward the caudal end in 10 mm increments. The x coordinate corresponds to the axial position measured in the cranio-caudal direction (see Figure 1). The stress was quantified via the median value of the peak von Mises stress recorded in 1 mm thick slices over the duration of the simulated event. Shading gives the stress distribution for the spine free of syringes. The lines display results for the following four scenarios: (**a**) syrinx expanding from S_1_ to S_1–4_, (**b**) syrinx expanding from S_1–5_ to S_1–10_, (**c**) syrinx expanding from S_1–11_ to S_1–18_, and (**d**) syrinx expanding from S_1–19_ to S_1–28_. Thick black lines correspond to the results for S_1–4_, S_1–10_, S_1–18_, and S_1–28_. Short vertical lines on the horizontal axis denote crania and caudal ends of the syrinx. Vertical broken lines divide the cord into cervical (C), thoracic (T), and lumbar (L) segment.

The next set of simulations examined the effect of an initial small syrinx at the cranial-most end of the spinal cord expanding caudally in 10 mm increments. It is worth reiterating that the syrinx radii for this set of models are the actual radii obtained from the MRI scans. The results are presented by focusing on the distribution of the median values of von Mises stress along the length of the spinal cord. This is illustrated in Figure 9, which has four parts (a–d). Each of the four plots covers a range of the syrinx expansion progression which exhibits a consistent pattern. As in Figure 7, the shaded area gives the stress values for the cord free of syringes (baseline values). The lines give the distribution of stress for all the configurations that exhibit the same pattern. The thick line corresponds to the configuration with the longest syrinx shown in a figure. Figure 9a shows the results for the syrinx expanding from 10 to 40 mm in length, i.e., starting with S_1_ and ending with S_1–4_. For this set of configurations, the stress is steadily increasing with the increasing length of the syrinx, reaching the value of 800 Pa for S_1–4_. The stress rises sharply toward the caudal end of the syrinx where it reaches its peak value and then suddenly drops. The stress in the remainder of the cord is not affected by the presence of the syrinx. Figure 9b shows the results for the syrinx expanding from 50 to 100 mm in length, i.e., starting with S_1–5_ and ending with S_1–10_. Here, the expansion of the syrinx does not lead to an increase in stress. The peak stress value is not at the caudal end of the syrinx but remains fixed at about 40 mm from the cranial end of the cord. With the syrinx expanding, the peak stress values actually drop, although they still remain above the baseline values. For S_1–10_, there is again a hint of a jump in stress at the caudal end of the syrinx. Figure 9c shows the results for the syrinx expanding from 110 to 180 mm in length, i.e., starting with S_1–11_ and ending with S_1–18_. In this region, the pattern where the peak value of stress is at the caudal end of the syrinx fully re-emerges. The peak stress reaches values of over 500 Pa. The stress is still elevated in the cervical region, but that local peak decreases with further expansion of the syrinx. For S_18_, the stress at 40 mm from the cranial end is slightly above the baseline value, whereas elsewhere in the cervical region the stress is below baseline values. Figure 9d shows the results for the syrinx expanding from 190 mm to its full length of 285 mm, i.e., staring with S_1–19_ and ending with S_1–28_. Here, the stress distribution changes from the previous in the sense that syrinx expansion has no effect on the cervical end of the spine. For the syrinx beyond 250 mm in length, the location of the peak stress is no longer at the caudal end of the syrinx, but rather remains locked at 250 mm from the cranial end of the cord. For the fully developed syrinx (S_1–28_), the stress is above the baseline values in the thoracic region. In the cervical region, it is below the baseline levels, except at a small region around 40 mm form the cranial end where it is slightly elevated. In general, for a fully developed syrinx, the stress values are moderate, and even if they are above the baseline values for a specific region of the cord (mainly thoracic), the values are always below 400 Pa.

## 4. Discussion

A semi-idealized computer model of the canine spinal column was used to simulate epidural excitation of CSF movement. Furthermore, 56 different syrinx configurations were considered to examine the effect of syrinx position and size on stress distribution in the spinal cord. In terms of the overall CSF dynamics, the results show that the rise in the epidural pressure is related to an influx of fluid (venous blood) into the epidural veins (the control volume, in the case of the model). That influx is minimal since all the tissues and fluids are nearly incompressible. Hence, the deformation of the dura and of the spinal cord are small. This explains why the stress in the cord is one order of magnitude lower than the pressure in the epidural space, SAS, and the syringes. For the most part, the movement of CSF and the movement of cord/syrinx are in the opposite directions. This motion pattern is governed by the “conservation of volume” in the spinal cavity. In comparison with the results of our previous study [36] where essentially the same model was subjected to cardiac pulse excitation imposed via CSF influx at the cranial end, the pressure in the fluids is one order of magnitude higher, but the CSF velocity and stress in the cord are of the same order of magnitude. Thus, although the epidural pressure is much higher than a normal CSF pulse pressure, it still creates roughly the same affect in terms of movement, deformation, and mechanical stress.

**Figure 10:**
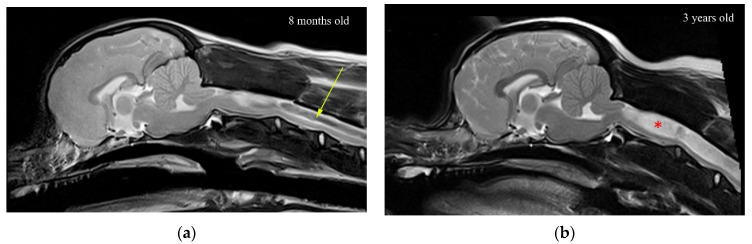
T2-weighted mid-sagittal MRI of the brain and cranial cervical spinal cord in CKCS with Chiari-like malformation and developing syringomyelia: (**a**) 8 months and (**b**) 3 years old. In (**a**), there is a (black) fluid signal-void sign within the center of the developing syrinx suggesting pulsatile or turbulent flow (arrow). In (**b**), the (white) syrinx has progressed to a large fluid-filled cavity within the spinal cord (red asterisk). There is less (black) fluid void sign suggesting less turbulent flow in this established syrinx.

The results clearly indicate that the stress in the region of a small syrinx is significantly higher than if the syrinx was absent. The magnitude of stress is dependent on the location of the syrinx. For an expanding syrinx, the situation is slightly more complicated. However, for most configurations, there was a significantly magnified stress at the caudal end of the syrinx. By observing the results for the movement of various anatomical layers (Figure 5a), we conclude that the small syringes and the surrounding cord deform in the longitudinal direction, thus generating stress in the spinal cord tissue. The deformation and stress at a syrinx are greater than elsewhere in the cord since the cord is “hollowed” and thus structurally more compliant. There is essentially no differential movement of fluid in the cavities with respect to the spinal cord tissue. Rather, they seem to move together. Strictly speaking, the slosh mechanism requires that fluid in the cavity moves at a different axial speed than the spinal cord tissue [30]. This results in the fluid being crammed into and expanding one end of a syrinx. This does not seem to happen in small, isolated syringes. In contrast, in larger syringes the motion of the fluid in the cavities is far more pronounced, and there is a clear differential movement of the fluid with respect to the spinal cord (Figure 5b). The bulk movement of the fluid is usually toward the caudal end of the syrinx where the highest values of stress are typically recorded for most configurations examined. Thus, it can be concluded that the model results support the slosh hypothesis for larger syringes. Figure 10 illustrates a developing and established syringomyelia in a cavalier King Charles spaniel. In the developing syrinx (a), when the dog is 8 months old, it can be appreciated that the center of the syrinx has a “core” of high velocity fluid illustrated by the “black” fluid void.

The experiment with small syringes indicates that the cord is the most sensitive to initial cavities when they are located in the cervical region, and in particular, at its cranial end (S_1_) and the cervico-thoracic junction (S_8_). These are roughly the locations where the initial cavities first form in CKCS dogs [38]. Thus, it may be argued that the initial syringes emerge at the locations where the cavities have the strongest effect on the amplification of stress in the cord. Assuming that the homeostatic mechanism applies, that will imply that the initial syringes are inherently unstable and that they will tend to expand. Conversely, small cavities should be the most stable if they are located in the thoracic region of the spinal cord. The results for the expanding syrinx suggest that the growth of an initial syrinx placed at the cranial end of the cord will initially result in the amplification of stress at the caudal end of the syrinx. Once the syrinx has reached the length of approximately 50 mm, the maximal stress is no longer at the caudal end but is locked at 40 mm from the cranial end. Further expansion of the syrinx will alleviate stress at that point, but eventually, the pattern where the stress peaks at the caudal end of the syrinx will reappear. It can be argued that the syrinx may stabilize in the cervical region if the stress is at tolerable levels throughout the cord for a certain syrinx length, or the amplification of stress at the caudal end will tip the balance, and the expansion will continue. Once the syrinx is 150 mm long. a typical pattern re-appears, with the peak stress at the caudal end. When the syrinx reaches its ultimate length, stress values become moderate in the sense that they are below the maximal values found in a cord free of syringes. Thus, it can be argued that a fully developed syrinx should be stable. This can be appreciated in Figure 10b, where there is a wide expanded syringomyelia, but the core of “black” high velocity fluid is no longer present.

Bilston et al. [25] postulate that a mismatch in the timing of CSF and arterial pulses may result in the CSF being forced from SAS into the spinal cord parenchyma via the perivascular space of the small blood vessels that penetrate the cord. This may happen against the pressure gradient due to the inertia of the fluid. We were not able to test whether this hypothesis holds in the case of CSF movement created by Valsalva, since that would require a different type of modeling, which focuses on perivascular spaces in a small segment of the cord. Another possibility is that repetitive exaggerated stressing of the cord at existing syringes alters the permeability of the pia membrane, thus further facilitating the movement of the fluid into the cavity. These hypothetical mechanisms deserve further attention in future studies.

We examined only one of the numerous possible scenarios for syrinx growth. For example, instead of choosing S_1_ configuration as the starting point, it was possible to choose another clinically plausible location for the initial syrinx. In addition, there could have been more than one small syrinx; the combination of S_1_ and S_8_ syringes would have been a clinically viable starting point. While this is technically trivial to accomplish, it would inevitably introduce an ambiguity in terms of the direction(s) in which syringes expand. Starting the syrinx from the cranial end meant that it could expand only caudally, which significantly reduced the number of scenarios that needed to be considered. It is more likely that a syrinx covering most of the cord length forms from a number of expanding initial small cavities. Such more complex scenarios of syrinx growth can be examined in future studies. Ideally, these should be based on detailed clinical history of the cavity expansion.

## 5. Conclusions

The results of this study strongly suggest that the spinal cord tissue in the vicinity of fluid-filled cavities experiences higher than normal mechanical stress due to the movement of the CSF from epidural excitation. When the syringes are longer than approximately 30 mm, filling of the epidural veins may generate the “slosh” effect, where the fluid is forced to the caudal end of the syrinx. The results for the simulations of an expanding syrinx are broadly consistent with the homeostatic hypothesis, as the stress in the cord is lower for the fully developed syrinx than for smaller syringes. Other, potentially more realistic, scenarios for syrinx expansion should be examined in the future. This study specifically addresses syringomyelia in dogs, and more specifically in CKCS. Since syringomyelia in humans and animals is essentially the same neurological disorder, the main conclusion that Valsalva may generate slosh should hold for humans as well as animals. Considering anatomical and other differences (e.g., upright posture in humans) the results regarding the potential pattern of syrinx enlargement do not apply to humans or to dog breads other than CKCS.

## Figures and Tables

**Table 1:** Material properties.

Spinal Cord	E = 62.5 kPa, ν = 0.49, *ρ* = 1000 kg/m^3^
Dura	E = 1.25 MPa, ν = 0.4, *ρ* = 1000 kg/m^3^
Epidural fat	E = 1 kPa, ν = 0.4999, *ρ* = 900 kg/m^3^
CSF and fluid in the syrinx	μ = 0.001 Pa s, *ρ* = 1000 kg/m^3^

E = Young’s modulus, ν = Poisson’s ratio, *ρ* = density, μ = dynamic viscosity.

## Data Availability

All data generated or analyzed during this study are included in this published article.

## Supplementary Materials

The following are available online at https://www.mdpi.com/article/10.3390/life11101083/s1, Geometric parameters of the model “Geometric parameters of the model.xlsx”.
